# Effects of Community-Based Cardiac Rehabilitation on Body Composition and Physical Function in Individuals with Stable Coronary Artery Disease: 1.6-Year Followup

**DOI:** 10.1155/2013/903604

**Published:** 2013-07-07

**Authors:** Sandra Mandic, Claire Hodge, Emily Stevens, Robert Walker, Edwin R. Nye, Dianne Body, Leanne Barclay, Michael J. A. Williams

**Affiliations:** ^1^Cardiac Rehabilitation Research Laboratory, School of Physical Education, Sport and Exercise Sciences, University of Otago, P.O. Box 56, Dunedin 9054, New Zealand; ^2^Department of Medicine, Dunedin School of Medicine, University of Otago, Dunedin 9054, New Zealand; ^3^Dunedin Hospital, Dunedin 9016, New Zealand

## Abstract

*Objective*. To examine long-term changes in physical function and body composition in coronary artery disease (CAD) patients participating in ongoing community-based cardiac rehabilitation (CR). *Design*. Thirty-four individuals (69.7 ± 8.2 years; 79% men) participated in this longitudinal observational study. Baseline and follow-up assessments included incremental shuttle walk, short physical performance battery, handgrip strength, chair stands, body composition, last year physical activity, and CR attendance. *Results*. Participants attended 38.5 ± 30.3% sessions during 1.6 ± 0.2 year followup. A significant increase in 30-second chair stands (17.0 ± 4.7 to 19.6 ± 6.4, *P* < 0.001), body weight (75.8 ± 11.1 to 77.2 ± 12.1 kg, *P* = 0.001), and body fat (27.0 ± 9.5 to 29.1 ± 9.6%, *P* < 0.001) and a decline in handgrip strength (36.4 ± 9.4 to 33.0 ± 10.6 kg·f, *P* < 0.001) and muscle mass (40.8 ± 5.6 to 39.3 ± 5.8%, *P* < 0.001) were observed during followup. There was no significant change in shuttle walk duration. CR attendance was not correlated to observed changes. *Conclusions*. Elderly CAD patients participating in a maintenance CR program improve lower-body muscle strength but experience a decline in handgrip strength and unfavourable changes in body composition, irrespective of CR attendance.

## 1. Introduction

Despite significant benefits associated with short-term (3 to 6 months) outpatient cardiac rehabilitation (CR) programs in coronary artery disease (CAD) patients, studies have consistently documented poor long-term adherence to physical activity recommendations [1, 2], worsening of cardiovascular risk factors [1, 3, 4], and a decline in exercise capacity [5] 12 to 18 months following the outpatient CR program. Several studies reported a decline in habitual physical activity during a long-term followup after hospital discharge [2] and after outpatient CR [1]. At 18 months after CR, only 27% of CAD patients adhered to minimal physical activity guidelines [1]. Considering that a great part of favourable hemodynamic, cardiorespiratory, and muscle strength adaptations are lost within 3 months of exercise cessation [6], it is essential for CAD patients to follow a regular and uninterrupted exercise program throughout life.

Strong evidence suggests that regular physical activity has favourable effects on body composition, lipoprotein profile, cardiovascular risk factors, and exercise capacity in individuals with cardiovascular disease [7]. Studies that have examined the effects of prolonged (>1 year) CR programs in cardiac patients found greater long-term compliance with physical activity recommendations [8, 9], favourable changes in body composition and lipoprotein profile [4, 10], less deterioration in body weight control [9], and maintenance or a slight additional increase in exercise capacity after the initial year of participation in CR program [10, 11]. Greater compliance with physical activity recommendations in CR participants can be at least in part attributed to social interaction, peer support, and supervision of CR programs. Therefore, preliminary evidence suggests that long-term CR with an exercise component may play an important role in slowing down the decline in exercise capacity and promoting independent living, particularly in elderly CAD patients. However, despite significant benefits including improvements in risk factors, [12], exercise capacity [12], physical function [13], and quality of life [12] and reduced mortality rates [14], elderly individuals with CAD are frequently not referred nor encouraged to attend outpatient CR programs [15]. In addition, logistic problems such as transport, cost, and limited availability of home-based or community-based CR programs further restrict CR participation among the elderly [15]. Therefore, long-term effects of CR in elderly individuals with CAD remain largely unknown. The purpose of this longitudinal observational study was to examine the long-term effects of CR attendance and physical activity habits on changes in body composition and physical function in elderly CAD patients participating in an on-going community-based maintenance CR program. 

## 2. Materials and Methods

### 2.1. Participants

Forty-six participants were recruited from two community-based CR programs in Dunedin, New Zealand (the Otago Phoenix Club and Taieri Fit and Fun Group) in 2009. Inclusion criteria were current membership at a community-based CR program (at least 1 group exercise session attended in 12 months before the baseline assessment) and a history of CAD (defined as a history of myocardial infarction, coronary artery bypass graft surgery, coronary angioplasty, or coronary stent insertion). Exclusion criteria were (1) a cardiac event (myocardial infarction, stent placement, or bypass graft surgery) or hospitalization with chest pain within the last 6 months; (2) chest pain coming on at rest; (3) significant palpitations; (4) moderate to severe aortic stenosis; and (5) significant dyspnoea and swelling. All participants were offered written and verbal information about the study and signed consent form. Ethics approval was granted by the Lower South Regional Ethics Committee and the University of Otago Ethics Committee. 

### 2.2. Study Design

Participants attended one visit at baseline (September to December 2009; *n* = 46) and followup (January 2011 to February 2012; *n* = 34) with an average followup of 1.6 ± 0.2 years and 26% loss to followup. The primary outcome measure was change in exercise capacity measured using a 10-meter shuttle walk test [16]. Secondary outcome measures included physical function, anthropometry, and body composition.

### 2.3. Community-Based Maintenance CR

Participants attended maintenance CR at one of the two local on-going community-based CR programs. Both programs offer two 60-minute group exercise sessions per week, consisting of a combination of aerobic, strength, flexibility, balance, and coordination exercises. All exercise sessions were led and supervised by physiotherapist or exercise specialist.

### 2.4. Measurement Procedures

#### 2.4.1. Medical History and Anthropometry

Self-reported demographics and medical history were obtained during an in-person interview at baseline. CAD history was verified through medical records. Height was measured using a stadiometer. Weight and body composition were measured using a bioimpedence scale (InBody 230, GBC BioMed NZ). Body composition assessment was completed before walking tests, at least 2 hours after the meal, and with a participant in light clothing and with bare feet. Participants were tested between morning and midday. InBody 230 device was placed horizontal to the ground and used in a research laboratory setting under normal temperature conditions. After ensuring proper feet placement and hold of hand electrodes, participants were instructed to maintain a normal standing position, with arms and legs extended, and stay relaxed and still during the measurements. Body mass index was calculated as weight (kg)/height (m^2^). Waist circumference was measured in standing position at the narrowest part of the torso (above the umbilicus and below the xiphoid process). Hip circumference was measured at the maximal circumference of the hip. Waist and hip circumferences were measured using a tape measure to the nearest 0.1 cm and waist-hip ratio was calculated (waist circumference/hip circumference). All measures were taken twice and averages were used in the analysis. 

#### 2.4.2. Shuttle Walk Test (see [16, 17])

 Participants walked up and down a 10-meter course around the cones placed at each end for up to 12 minutes, as described previously [17]. The predetermined walking speed was dictated by an audio signal starting at 1.8 km/h and was progressively increased by ~0.6 km/h each minute up to 8.5 km/h at the end of the test. The participant was required to reach the cone before the next beep. Heart rate and rate of perceived exertion were recorded every minute and after test. The test was terminated when participants could not maintain the pace or experienced symptoms of exercise intolerance [17].

#### 2.4.3. Short Physical Performance Battery

Physical function was assessed using a validated and standardized short physical performance battery [18]. The assessment consisted of progressive balance tests (up to 10 seconds for 3 different foot positions), gait speed test (timed “usual pace” 4-meter walk), and repeated chair stands (time to complete 5 consecutive sit to stands). Each of these measures was scored on a scale 0 (incomplete test) to 4 (highest performance). A summary score ranging from 0 (poor function) to 12 (excellent function) was calculated by adding the 3 individual scores together.

#### 2.4.4. Muscle Strength

Upper-extremity muscle strength was assessed using a handgrip dynamometer (Lafayette Model 78010). In a sitting position, with the feet flat on the floor, the participant held a handgrip dynamometer parallel to the floor with the wrist in line with their elbow and was instructed to squeeze the dynamometer as hard as possible. The test was repeated 3 times on each hand. The handgrip strength index was calculated as the average of the highest value of the right and left handgrip strength [19]. Lower-extremity muscle strength was assessed using a 30-second chair stand test [20] performed twice and with the higher score used in the analysis.

#### 2.4.5. Physical Activity

 Self-reported physical activity habits over the past 12 months were assessed in an interview. Participants recalled the mode, frequency, duration, and self-perceived intensity (“light”, “somewhat, hard”, or “very hard”) of each physical activity they had regularly participated in over the previous 12 months taking into account seasonal variations (months per year). Metabolic equivalents (METs) of each activity were determined from the Physical Activity Compendium [21]. The total amount of time spent in each moderate-to-vigorous physical activity (≥3.0 METs) multiplied by corresponding METs for each activity was used to calculate energy expenditure in kilocalories using the following equation: kilocalories·h^−1^ = MET × weight (kg) × duration (hours) [21] and expressed as the average weekly energy expenditure (kcal/week). Based on average weekly energy expenditure, participants were categorized as sedentary (<1000 kcal/week), moderately active, (1000–1999 kcal/week), and active (≥2000 kcal/week) [22]. To reduce a potential bias for greater energy expenditure in kilocalories in individuals with a higher body weight, energy expenditure was also expressed as METs-minutes per week.

#### 2.4.6. CR Attendance

 Attendance records were acquired from both clubs to calculate membership duration (years since the first attended session) and the percentage of sessions attended between baseline and follow-up assessments. 

### 2.5. Statistical Analysis

Descriptive statistics was used to analyse participant demographics, medical history, and medications. Analysis of changes between baseline and follow-up assessments was performed using Repeated Measures ANOVA for continuous variables and a chi-square test for categorical variables. Partial correlation tests (controlling for age and gender) were used to examine the correlations between CR attendance and last year physical activity with continuous outcome variables. *P *value <0.05 was considered statistically significant. Data were analysed using SPSS Statistics Version 19 statistical package.

## 3. Results

A total of 46 individuals with CAD completed baseline assessment and 34 (74%) participants returned for followup testing 1.6 ± 0.2 years later (range: 1.3 to 2.4 years). The twelve (26%) individuals who were lost to followup tended to be older (74.9 ± 9.3 versus 69.7 ± 8.2 years, *P* = 0.076) and have reduced exercise capacity at baseline (shuttle walk test duration: 7.8 ± 2.1 versus 9.0 ± 1.9 min, *P* = 0.071) compared to individuals who completed both assessments. All data presented in this report are based on the 34 participants who completed both assessments.

The majority of the participants were married, retired elderly males, with less than university education (Table 1). CAD history, and medications are presented in (Table 1). CAD history included myocardial infarction (22 (64.7%)), angina (20 (58.8%)), coronary artery bypass surgery (11 (32.4%)), angioplasty (4 (11.8%)), and stents (10 (29.4%)) (Table 1). The average time since last CAD event was 5.6 ± 6.2 years (range 0.5 to 33.7 years). Other cardiovascular history included valvular surgery (2 (5.9%)), peripheral vascular disease (1 (2.9%)), heart failure (1 (2.9%)), transient ischemic attack (1 (2.9%)), and stroke (1 (2.9%)). Risk factors included hypertension (15 (44.1%)), dyslipidemia (24 (70.6%)), smoking (8 (23.5%)), obesity (5 (14.7%)), diabetes (3 (8.8%)), and family history of cardiovascular disease (13 (38.2%)). On average, participants had 1.6 ± 1.2 modifiable cardiovascular risk factors. Other chronic medical conditions included musculoskeletal problems (22 (64.7%)), cancer (6 (17.6%)), asthma (1 (2.9%)), anxiety (4 (11.8%)), and depression (4 (11.8%)).

The average CR membership was 3.2 ± 1.6 years (range 0.4 to 5.3 years) with attendance at CR sessions (held twice per week) of 38.5 ± 30.3% during the followup period. Seven (20.6%) participants did not attend any CR sessions while 11 (32.4%) of participants attended at least 50% of the sessions during the followup. Based on self-reported 12-month physical activity, 10 (29.4%) of participants were categorized as sedentary, 6 (17.6%) were moderately active, and 18 (52.9%) were considered active at followup. The average energy expenditure in physical activity over the previous 12 months was 2249 ± 1714 kcal/week and 32.0 ± 31.0 METs-hours per week. Self-reported 12-month physical activity did not correlate to CR attendance (*r* = −0.01; *P* = 0.962).

During the followup, there were significant changes in anthropometry (increased body weight, body mass index, and waist circumference) and body composition (reduced percentage of muscle mass and increased percentage of body fat) (Table 2). A small but significant reduction in lean mass was observed in lower extremities (from 16.6 ± 2.9 kg to 16.2 ± 2.9 kg; *P* = 0.007). A significant improvement in number of completed chair stands and a decline in handgrip strength were observed during the followup. Physical function did not change significantly.

After accounting for potential effects of age and gender, the observed changes in anthropometry and body composition did not correlate to CR attendance or self-reported physical activity during the followup (data not presented). Higher levels of self-reported physical activity at the followup were significantly correlated to the shuttle walk duration (*r* = 0.518, *P* = 0.002) and higher number of chair stands (*r* = 0.488. *P* = 0.005). CR attendance was not related to exercise capacity, muscle strength, physical function, or anthropometry measures at the followup assessment.

## 4. Discussion

Previous studies have consistently documented poor long-term adherence to physical activity recommendations [1], worsening of cardiovascular risk factors [1], and decline in exercise capacity [5] 12 to 18 months following outpatient (phase II) CR program. This prospective observational study examined the effects of long-term attendance to on-going community-based maintenance CR and self-reported physical activity habits on changes in body composition and physical function in CAD patients. During a 1.6-year followup, CAD patients experienced a significant increase in lower-body muscle strength, body weight, and body fat and a decline in muscle mass and handgrip strength, irrespective of CR attendance rates and self-reported physical activity in the previous year. Exercise capacity and physical function remained unchanged during the followup. CR attendance did not correlate to self-reported physical activity in the previous year. However, higher levels of physical activity in the last 12 months were associated with higher exercise capacity and lower-body muscle strength at the follow-up assessment.

Aging is associated with unfavourable changes in body composition, including increased body fat and reduced muscle mass [23]. Middle-aged CAD patients participating in an extended CR program had favourable changes in body composition beyond changes observed during the initial 3-month outpatient CR [10]. Participants showed a smaller increase in body weight and greater decrease in percentage of body fat compared to nonparticipants [10]. In the present study, both body weight and body fat increased and muscle mass decreased during the 1.6-year followup in an older sample of individuals with CAD. These changes were not correlated to CR attendance or self-reported physical activity in the previous year. Therefore, ageing as well as other factors contribute to weight gain and unfavourable body composition changes in older CAD patients, irrespective of participation in a maintenance CR program. 

Attendance to short-term outpatient CR following cardiac event improves physical function [13, 24] and exercise capacity [12] in elderly CAD patients. After the initial year of CR, long-term participation in CR programs results in maintenance or slight additional increase in exercise capacity in middle-aged CAD patients [10, 11]. Brubaker et al. reported a 6% increase in exercise capacity (estimated METs) following 3-month phase II CR in 25 CAD patients attending an extended CR program (average 2.5 years (range 1–5 years)). Rogers et al. reported a 44% increase in measured exercise capacity (peak oxygen consumption) in 9 CAD patients after 12-month exercise training with no further increase in exercise capacity observed after 6 additional years of intense exercise training [11]. Both of these studies were performed in middle-aged individuals. The present study extends previous findings by reporting no significant change in exercise capacity or physical function observed in older CAD patients participating in long-term community-based CR on average 5 to 6 years after their last cardiac event. Therefore, long-term maintenance CR programs with an exercise component may play an important role in maintaining and/or slowing down the decline in exercise capacity, rather than improving it beyond increases gained during the first year postcardiac event. Slowing down the decline in exercise capacity and physical function is particularly important for elderly individuals to prolong independent living. 

Muscle strength is a predictor of physical function in elderly individuals [25] and correlates with 6-minute walk test distance [26] and peak oxygen consumption [27] in CAD patients. In the present study, CAD patients showed a significant improvement in lower-body muscle strength but a decline in handgrip strength. Age-associated decline in muscle strength is likely to be due to a combination of sarcopenia [28] and/or deconditioning. In the present study, muscle mass was significantly reduced at the 1.6-year followup assessment in elderly CAD patients. Therefore, muscle strengthening exercises, particularly for the upper body, should be emphasized as a part of long-term maintenance CR programs for elderly individuals.

Previous studies reported poor long-term adherence to physical activity recommendations 1 to 1.5 years following outpatient CR program in individuals with CAD [1]. However, participation in structured maintenance CR programs is associated with a higher likelihood of regular physical activity following outpatient CR in individuals with CAD [8] and coronary artery bypass surgery [29]. In the present study, 71% of participants were considered moderately active or active at the followup assessment. Over half of participants (55.9%) exceeded the minimal energy expenditure threshold of 1,800 kcal/week for slowing the progression of coronary artery stenosis [30]. Interestingly, self-reported physical activity in the previous year was not correlated to attendance of community-based CR sessions. In addition, neither CR attendance nor self-reported physical activity correlated with observed changes in weight, and body composition, or handgrip strength during the followup. A dose-response relationship has been reported between attendance to outpatient CR and long-term survival in elderly individuals CAD [31], suggesting that attendance to outpatient CR may be a proxy for other factors including health status [32]. In the present study, participants on average attended 38.5% of community CR exercise sessions (held twice weekly) which is equivalent to approximately 1 exercise session per week. This amount of exercise is not sufficient to obtain health benefits from CR participation. Previous studies reported an average energy expenditure of 230 kcal per exercise session [33] and 1,504 kcal/week [34] in CAD patients participating in maintenance CR with 72% participants meeting minimal physical activity guidelines [34]. Similarly, in the present study, 71% of participants met minimal physical activity guidelines when overall physical activity was taken into account. However, these data should be interpreted with caution due to the inaccuracy of physical activity recall, particularly in elderly individuals. 

This study has several limitations including a small sample size, lack of a control group, and 26% loss to followup. In addition, the results of this study are based on a selected population. We do not have information on the percentage of eligible candidates who were not referred to, refused to participate in, or dropped out of these programs. Similarly, we do not have information on participants' health status after the cardiac event. As suggested previously for outpatient CR [31], low risk individuals may not feel the need to attend CR and/or high risk individuals may not be able to tolerate CR. Therefore, health status after a cardiac event and participation in outpatient CR may be a significant determinant of participants' perceived need and ability to join community-based CR. In addition, self-reported physical activity is prone to recall and social desirability biases and may be challenging for elderly individuals. The short physical performance battery used for assessment of physical function may not have been sensitive enough to detect small changes among well-functioning elderly individuals in the present study. Finally, followup duration may not have been long enough to observe significant changes in physical function in this well-functioning selected sample of CAD patients. Future studies should use objective measures of physical activity, recruit age- and gender-matched participants from the community as a control group, and examine the effects of community-based CR on psychosocial health to determine factors associated with long-term adherence to these programs. The examination of the long-term effect of community-based maintenance CR will likely remain challenging due to scarce availability of such programs, poor compliance with long-term exercise interventions, selection bias associated with a long-term adherence to a structured program, and nonfeasibility of randomized controlled trials.

## 5. Conclusions

In conclusion, elderly CAD patients participating in long-term community-based CR programs improved lower-body muscle strength and experienced a decline in handgrip strength and unfavourable changes in body composition during 1.6-year followup. These changes were observed irrespective of CR attendance and self-reported physical activity in the previous year. Exercise capacity and physical function did not change significantly. Therefore, community-based CR programs may play an important role in long-term maintenance and/or slowing down the decline in lower-body muscle strength which is particularly important for maintenance of independent living in older CAD patients. These programs should also emphasize upper-body muscle strength exercises and encourage participants to engage in additional physical activity outside of the community-based CR sessions. 

## Figures and Tables

**Table 1:** Demographic characteristics.

Variable	Total sample n = 34

Age (years)	69.7 ± 8.2
Males/females [*n* (%)]	27 (79.4)/7 (20.6)
Ethnicity: New Zealand European [*n* (%)]	31 (91.2)
University education [*n* (%)]	11 (32.4)
Married [*n* (%)]	26 (76.5)
Retired [*n* (%)]	26 (76.5)
Medical history [*n* (%)]	
Myocardial infarction	22 (64.7)
Angina	20 (58.8)
Coronary artery bypass graft surgery	11 (32.4)
Coronary artery stent	10 (29.4)
Coronary artery angioplasty	4 (11.8)
Medications [*n* (%)]	
Beta-blockers	27 (79.4)
ACE-inhibitors	9 (26.5)
Calcium channel blockers	3 (8.8)
Aspirin	28 (82.4)
Lipid lowering drugs	29 (85.3)
GTN spray	1 (2.9)
Nitrates	2 (5.9)
Diuretics	3 (8.8)

ACE: Angiotensin converting enzyme; GTN: Glyceryl trinitrate.

**Table 2:** Changes in anthropometry, body composition, and physical function during 1.6-year followup.

Variable	Baseline *n* = 34	Followup *n* = 34	*P*-value
Anthropometry			
Weight (kg)	75.8 ± 11.1	77.2 ± 12.1	0.001
Body mass index (kg/m^2^)	26.8 ± 3.5	27.3 ± 4.0	0.002
Waist circumference (cm)	92.0 ± 9.1	95.2 ± 10.6	0.002
Hip circumference (cm)	104.7 ± 18.8	103.5 ± 8.4	0.673
Waist to hip ratio	0.89 ± 0.1	0.92 ± 0.1	0.046
Body composition			
Body fat (%)	27.0 ± 9.5	29.1 ± 9.6	0.000
Muscle mass (%)	40.8 ± 5.6	39.3 ± 5.8	0.000
Physical function			
Shuttle walk test (min)	9.0 ± 1.9	8.8 ± 1.8	0.392
Short physical performance	11.59 ± 1.0	11.59 ± 0.9	0.999
Battery (0–12)			
Handgrip strength (kg·f)	36.4 ± 9.4	33.0 ± 10.6	0.000
30-second chair stands (*n*)	17.0 ± 4.7	19.6 ± 6.4	0.001
